# Hybrid Pickering emulsifiers (HYPIEs) via synergistic water/oil interfacial interactions: enhanced properties and applications

**DOI:** 10.1093/nsr/nwag149

**Published:** 2026-03-12

**Authors:** Jean-François Dechézelles, Yaoyao Feng, Kang Wang, Marc Pera-Titus, Véronique Nardello-Rataj

**Affiliations:** CNRS, UMR 8181-UCCS-Unité de Catalyse et Chimie du Solide, Université de Lille, Lille 59000, France; CNRS, UMR 8181-UCCS-Unité de Catalyse et Chimie du Solide, Université de Lille, Lille 59000, France; Cardiff Catalysis Institute, School of Chemistry, Cardiff University, Cardiff CF10 3AT, UK; Cardiff Catalysis Institute, School of Chemistry, Cardiff University, Cardiff CF10 3AT, UK; CNRS, UMR 8181-UCCS-Unité de Catalyse et Chimie du Solide, Université de Lille, Lille 59000, France

**Keywords:** Pickering emulsion, hybrid Pickering emulsifier, surface-active particles, synergy, interface

## Abstract

Emulsions stabilized by surface-active solid particles, known as Pickering emulsions, offer advantages over conventional emulsions, including enhanced stability, biocompatibility and the potential recyclability of particles. These attributes underpin their growing use in cosmetics, food, catalysis, enhanced oil recovery and pharmaceuticals. However, single-particle emulsifiers often lack the versatility required to precisely control emulsion formation, stability, and morphology. To address these limitations, *hybrid Pickering emulsifiers* (HYPIEs), which combine solid particles with secondary emulsifiers, have emerged as a powerful alternative. By improving interfacial wettability and adsorption, HYPIEs exhibit superior performance compared with their individual components and enable the co-adsorption of species with antagonistic properties, facilitating the design of smart emulsifying systems. This review categorizes HYPIEs based on synergistic combinations, including particle–particle (i.e. ‘hard’–‘hard’, ‘hard’–‘soft’, and ‘soft’–‘soft’), particle–(bio)surfactant and particle–(bio)polymer systems. We elucidate the mechanisms underlying their enhanced interfacial behavior and highlight the diverse interfacial architectures that can be achieved. Finally, we discuss emerging applications of HYPIEs in shaping oil–water interfaces for catalysis, biomedicine, cosmetics, personal care and food products, and outline current challenges and future perspectives, including strategies for tailoring synergistic functionalities and computational approaches for *in silico* HYPIE design.

## Teaser

Hybrid Pickering emulsifiers combine different particles or particles with surfactants and polymers to create smarter, more stable oil–water mixtures, enabling broader applications in food, cosmetics, medicine, and catalysis.

## INTRODUCTION

Emulsions are widely used in industrial processes and commercial products due to their versatile functionality across multiple sectors [1]. Emulsions are typically stabilized by surfactants that adsorb at the oil–water interface, reducing interfacial tension and preventing phase separation. However, surfactants have drawbacks, including environmental contamination, recovery challenges and potential microbial degradation [2]. Increasing demands for biocompatibility—especially in food and biomedical applications—further limit the use of synthetic surfactants.

Unlike molecular surfactants, solid particles adsorb at the oil–water interface based on *dual wettability*, i.e. their affinity for both oil and water phases. Droplet size, emulsion stability and type are determined by particle wettability [3,4]. Pickering emulsions, like all emulsions, can be classified as oil-in-water (O/W) or water-in-oil (W/O), depending on the wettability of the solid particles at the oil–water interface. When particles are more hydrophilic (contact angle *θ* < 90°), they preferentially stabilize O/W emulsions, whereas more hydrophobic particles (*θ* > 90°) preferentially stabilize W/O emulsions [5]. The continuous phase is the liquid wetting the particles more.

Physicochemical parameters such as particle size, shape, surface roughness, surface chemistry and intra/interparticle porosity further influence hydrophilic/hydrophobic balance and so, emulsifying efficiency [6–8]. Solid particles offer additional advantages, allowing tailored functionalities and facile recovery via centrifugation, salt addition or thermal treatment.

Surface-active particles stabilize emulsions through a combination of electrostatic, van der Waals, hydrophobic, steric, capillary and depletion forces [9,10], forming a physical barrier against droplet coalescence [11,12]. The balance of these forces governs particle–interface and particle–particle interactions. Hydrophobic interactions can be enhanced by surfactants, which suppress electrostatic repulsions at lower interfacial charge [13], and can synergistically reduce surface tension [14]. Steric stabilization arises when polymer chains or bulky molecules adsorb on particles, creating repulsive interactions from the entropic penalty of compressing overlapping chains; this operates over longer distances than electrostatic forces [15,16]. Capillary interactions between particles enhance mechanical rigidity through interfacial deformation [17], and can be tuned by additives (e.g. surfactants, polymers) that modify wettability and interfacial tension.

Surface engineering of particles often demands complex, expensive or toxic reagents. To optimize formation, stability and morphology of Pickering emulsions, single-particle emulsifiers may be insufficient. A promising alternative is the use of co-emulsifiers that interact synergistically with particles at the oil–water interface to form *hybrid Pickering emulsifiers* (HYPIEs). These ‘smart’ hybrids can modulate the interfacial microenvironment, enabling cooperative interactions that exceed the sum of individual contributions, facilitating the design of custom-made emulsions with enhanced stability, tunable morphology, inversion control and tailored interfacial properties.

This review presents a taxonomy of synergistic interactions in HYPIEs, including modifications in wettability, colloidal tectonics, local structural reconfiguration and viscoelasticity enhancement. Based on this taxonomy, we classify HYPIEs as combinations of two particles (‘hard’–‘hard’, ‘hard’–‘soft’, ‘soft’–‘soft’; hard = inorganic particle, soft = folded globular (bio)polymer, protein particles, solid wax, surfactant micelles), one particle and a surfactant, or one particle and an unfolded (bio)polymer. Selected examples of HYPIEs for applications in catalysis, enhanced oil recovery (EOR) and biomedicine are then listed. The final section lists the conclusions and addresses the future directions and engineering prospects of HYPIEs.

The terms ‘hard’ and ‘soft’ particles are used in an interfacial-mechanical sense rather than a purely chemical one. ‘Hard’ particles refer to colloids that remain mechanically rigid and non-deformable at the oil–water interface, including inorganic particles as well as non-swellable polymer latexes, e.g. polystyrene or poly(methyl methacrylate) particles. In contrast, ‘soft’ particles denote deformable, swellable or conformationally adaptive entities—such as protein microgels, polysaccharide microgels, wax crystals or surfactant micelles—that can reorganize, partially unfold, or deform upon interfacial adsorption. This distinction is essential for understanding how particle mechanics influence interfacial packing, film elasticity and emulsion stability.

## TAXONOMY OF SYNERGISTIC INTERACTIONS FOR ENGINEERING HYPIES

**Figure 1.**
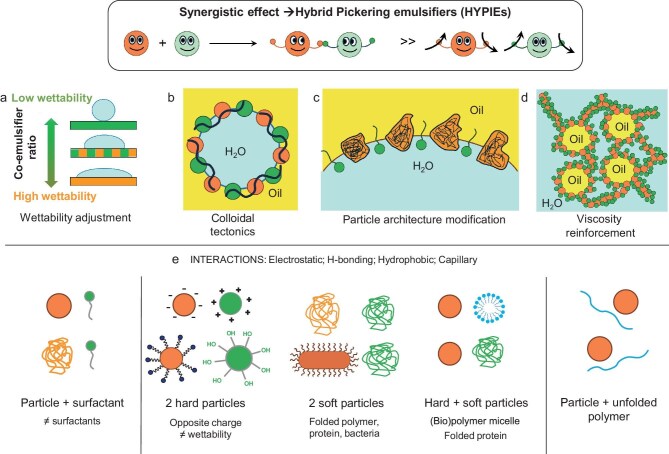
Taxonomy of synergistic interactions to design HYPIEs for stabilizing Pickering emulsions with adjustable properties: (a) wettability adjustment; (b) colloidal tectonics; (c) particle architecture modification; (d) viscosity reinforcement; (e) interactions.

HYPIEs can be engineered at the oil–water interface by leveraging four main principles, depending on the nature and strength of intermolecular forces (Fig. 1):

- Wettability adjustment (Fig. 1a). Co-emulsifiers can tune particle wettability to match a specific oil–water system. This occurs through adsorption of pairs of ‘hard’ or ‘soft’ hydrophilic/hydrophobic particles, particles with opposite charges, or combinations of particles with surfactants or polymers, forming hetero-aggregates. These interactions are driven by electrostatic, hydrogen-bonding and hydrophobic forces.
- Colloidal tectonics (Fig. 1b). Synergy can arise from structural interlocking between porous particles and co-emulsifiers via van der Waals forces (often involving a second particle type), forming stable biparticle assemblies at the interface. This architecture-driven organization reinforces interfacial stabilization.
- Particle architecture modification (Fig. 1c). Co-emulsifiers can induce conformational changes in particle structure at the interface through electrostatic, hydrogen-bonding, hydrophobic and depletion forces. For example, folding or unfolding of protein (‘soft’) particles promotes tighter interfacial packing, enhancing emulsion stability.
- Viscoelastic reinforcement (Fig. 1d). Synergistic interactions can alter the viscoelastic properties of the continuous phase, sometimes generating gel-like behavior. Dense interfacial layers and particle networks at droplet surfaces inhibit coalescence and suppress creaming, extending emulsion lifetime. Such networks are common in HYPIEs combining two particles (‘hard’ or ‘soft’) with opposite charges.

Importantly, particle ‘softness’ in HYPIEs should be understood as an emergent interfacial property—governed by deformability, swelling and conformational freedom—rather than by chemical composition alone.

## HYPIES BASED ON THE SYNERGY BETWEEN TWO PARTICLES

Combining two distinct particles with different functional groups, charges or wettability can generate HYPIEs at the oil–water interface, enhancing emulsification [18]. Such HYPIEs can involve two ‘hard’ (inorganic) particles, one ‘hard’ and one ‘soft’ (organic, macromolecular) particle, or two ‘soft’ (organic, macromolecular) particles (Fig. 1e). Selected seminal examples are summarized below.

### HYPIEs based on the synergy between two ‘hard’ particles

Dual-particle systems composed of inorganic (‘hard’) particles with differing wettability or charge can co-adsorb at the interface, increasing interfacial coverage, modulating emulsion type and forming particle networks that enhance viscosity, stability and allow transitional inversion.

#### Synergy between two ‘hard’ particles with different wettability

**Figure 2.**
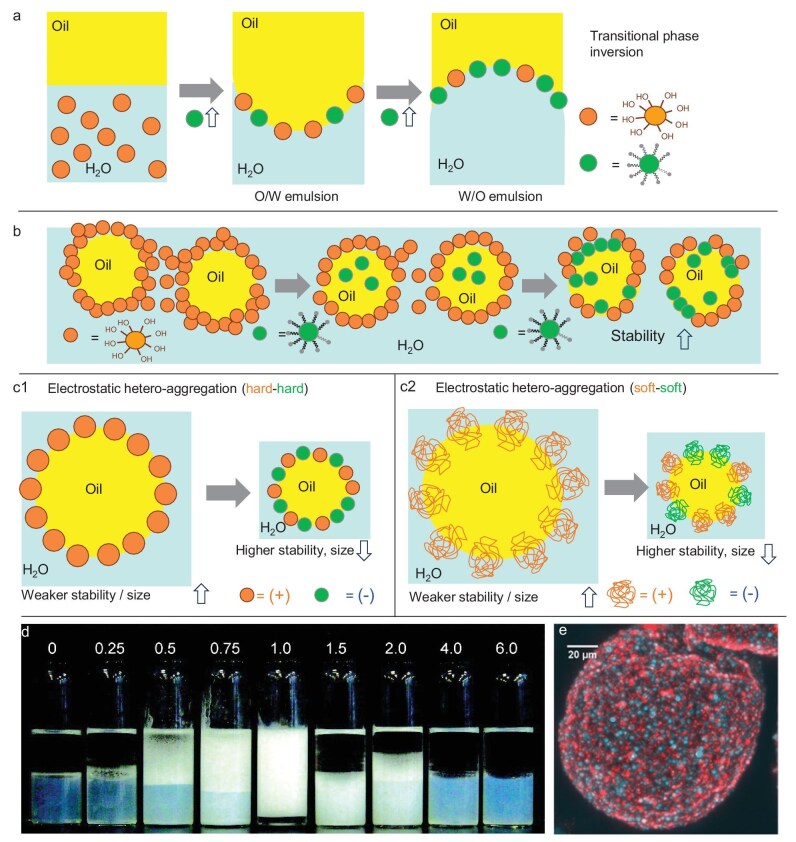
(a) HYPIEs combining two ‘hard’ hydrophilic and hydrophobic particles showing transitional phase inversion as a function of the particle ratio [19]. (b) Schematic representation of the interfacial structure of O/W emulsions stabilized by hydrophilic particles or mixed hydrophilic/hydrophobic particles [20]. (c1 and c2) Emulsion droplet stabilized by oppositely charged silica/alumina (hard–hard) and polymeric (soft–soft) particles: cationic (orange) and anionic (green), 50/50 w/w [21,22,24]. (d) Photographs showing the appearance of dodecane–water mixtures (1:1 by volume) after 24 h stabilization by a combination of 2 wt% LUDOX HS-30 particles and LUDOX CL particles at varying initial concentrations (wt%). Stable O/W emulsions were observed only at intermediate concentrations. At 1 wt%, the emulsions exhibited a gel-like consistency with minimal creaming and no coalescence (reproduced from Binks *et al*. [21] with permission from the Royal Society of Chemistry). (e) Confocal laser scanning fluorescence microscopy image of an emulsion droplet stabilized by a mixture of cationic polymeric particles (in red) and anionic polymeric particles (in blue), 50/50 w/w (reproduced from Ref [24] with permission from the Royal Society of Chemistry).

Binks and Lumsdon [19] first demonstrated synergistic effects between hydrophilic and hydrophobic silica particles in water–toluene systems (Fig. 2a). Increasing the proportion of hydrophilic silica induced a transitional inversion from W/O to O/W emulsions, while excess hydrophobic particles (>4:2 w/w) caused droplet deflocculation. Emulsion stability is strongly influenced by the spatial arrangement of hydrophilic and hydrophobic particles at the oil–water interface. For example, Aerosil^®^R7200 (hydrophilic) and Aerosil^®^R972 (hydrophobic), when used individually, form network-like interfacial structures that impart gel-like rheological properties (Fig. 2b) [20]. In contrast, mixtures with high hydrophobic-to-hydrophilic ratios exhibit lower viscosity and reduced stability, as hydrophobic particles preferentially orient toward the oil phase, weakening the interfacial network.

#### Synergy between two ‘hard’ particles with opposite charge

Oppositely charged ‘hard’ particles (e.g. anionic silica + cationic alumina) can form stable HYPIEs at the oil–water interface, leading to O/W emulsions and gels (Fig. 2c1) [21,22]. Electrostatic attraction between particles can reduce the net surface charge facilitating hetero-aggregation and rapid interfacial assembly. The resulting particle networks spreading over the continuous phase can stabilize emulsions for over a year and serve as templates for porous ceramics (see representative emulsions in Fig. 2d).

Nallamilli and co-workers [23] reported the first example of O/W emulsions stabilized by mixtures of oppositely charged polystyrene (PS) particles. Although composed of polymeric material, these PS particles behave as rigid, non-swellable colloids under the studied conditions and therefore fall within the category of mechanically ‘hard’ particles at the oil–water interface. Aggregates initially formed in the aqueous phase and subsequently rearranged into dense monolayers at the interface, stabilizing droplets. Adjusting the oil-to-water ratio induced catastrophic phase inversion. Synergistic effects were also observed using combinations of three types of poly(methyl methacrylate) (PMMA) particles with neutral, anionic and cationic surface charges [24]. Anionic particles achieved much higher droplet coverage (∼90%), due to faster interfacial adsorption. In mixtures of neutral + anionic or neutral + cationic particles, droplet coverage varied linearly between that of the corresponding single particles, accelerating overall adsorption. Combining anionic and cationic particles with neutral particles produced smaller droplets and higher particle coverage. In emulsions combining anionic and cationic particles, hetero-aggregates formed in the bulk leading to emulsions with very small droplet sizes via a particle network (Fig. 2c2 and e).

### HYPIEs based on the synergy between two ‘soft’ particles

Two ‘soft’ particles (e.g. folded proteins or polymer particles) can stabilize food-grade emulsions. Their folding or unfolding depends on intramolecular interactions and solvent polarity. Enzyme-based protein particles are discussed separately later. HYPIEs can be designed by combining positively charged ‘soft’ zein and negatively charged ‘soft’ pectin particles driven by electrostatic interactions to form stable high-internal phase emulsions, modulating interfacial self-assembly [25].

### HYPIEs based on the synergy between one ‘hard’ and one ‘soft’ particle

**Figure 3.**
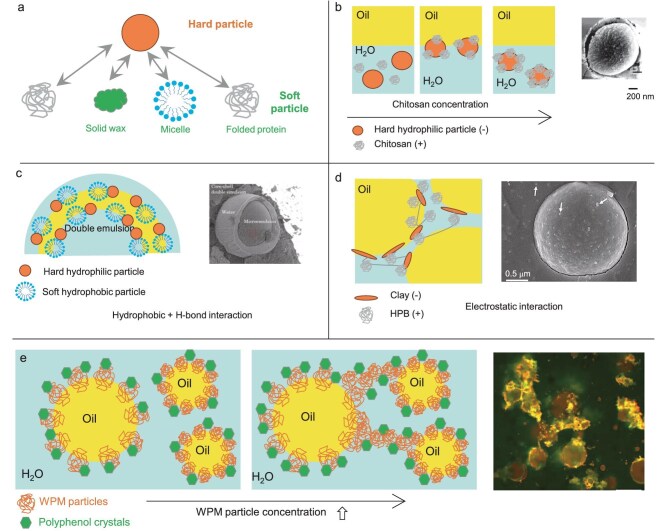
(a) Schematic representation of hard and soft particles. (b) Emulsion stabilized by HYPIEs based on a combination of ‘hard’ hydrophilic silica particles and ‘soft’ CS particles [26], and cryo-SEM picture of an O/W droplet stabilized by the combination of silica particle and CS (reproduced from Alison *et al.* [26] with permission from the American Chemical Society). (c) Double emulsion stabilized by ‘hard’ hydrophilic silica particles and surfactant micelles [27,28], and cryo-SEM of the double emulsion (reproduced from Bazazi *et al.* [28] with permission from Elsevier). (d) Self-supporting 3D hydrophobin–clay network and cryo-SEM image of an emulsion droplet [29]; HPB molecules, clay and the interaction between HPB are represented as a gray linear loop, orange oval and gray line, respectively (reproduced from Reger *et al.* [29] with permission from the Royal Society of Chemistry). (e) Interfacial arrangement of ‘hard’ polyphenol crystals and ‘soft’ WPM particles, and confocal image of emulsion stabilized by a polyphenol (curcumin) and WPM (reproduced from Zembyla *et al.* [30] with permission from the American Chemical Society). At low WPM concentration, both components co-adsorb at the interface; at higher WPM concentration, particle aggregates form interfacial and inter-droplet bridges [30].

Combining ‘hard’ and ‘soft’ particles—where ‘soft’ refers to deformable or interfacially adaptive entities such as folded proteins, polymer microgels, wax crystals or surfactant micelles—stabilizes emulsions through electrostatic, hydrogen-bonding and hydrophobic interactions (Fig. 3a).

#### Synergy between one ‘hard’ particle and one ‘soft’ (bio)polymer particle

HYPIEs can be generated by assembling negatively charged hydrophilic silica particles with non-covalently bonded positively charged chitosan (CS) bio-oligomer particles via electrostatic interactions (Fig. 3b) [26]. Partial hydrophobization of silica particles promoted interfacial adsorption, resulting in O/W emulsions with stability longer than 3 months. Low CS concentrations (1 wt% relative to silica) enhanced interfacial viscoelasticity, whereas higher CS content (5 wt%) promoted droplet-surrounding networks.

#### Synergy between one ‘hard’ particle and one ‘soft’ wax or surfactant micelle particle

HYPIEs can be generated combining laponite and wax crystals driven by hydrogen-bonding and hydrophobic interactions (Fig. 3c) [27]. Wax crystals can protrude through the oil–water interface from oil droplets, promoting coalescence. During emulsification at elevated temperatures, wax remains liquid-like, but upon cooling, wax crystals can form around the interface, enhancing viscoelasticity that provides a physical barrier against coalescence. Bazazi and Hejazi [28] extended this concept to combinations of hydrophilic silica particles and Span-type surfactant micelles, with hydrophobic and hydrogen-bond interactions being key drivers of silica–Span interfacial co-adsorption.

#### Synergy between one ‘hard’ particle and one ‘soft’ folded (globular) protein particle

HYPIEs based on laponite and Protein B (HPB or hydrophobin) particles can stabilize emulsions via electrostatic assembly of protein-coated laponite into a 3D interfacial network that produced gel-like O/W emulsions (30–65 wt% oil) (Fig. 3d) [29]. This laponite–HPB network enhanced the elastic properties of the gels and improved emulsion stability.

Also, HYPIEs based on hydrophobic, water-insoluble ‘hard’ anionic polyphenol crystals and cationic whey protein microgel (WPM) particles can adsorb from the oil and aqueous side, respectively, providing double interfacial stabilization (Fig. 3e) [30]. This synergy formed an interfacial viscoelastic film with droplets fully covered by a dense monolayer of WPM particles, with WPM acting as a ‘colloidal glue’ between polyphenol crystals and water droplets stabilizing droplets at higher concentrations. Likewise, HYPIEs based on cellulose nanocrystal (CNC) particles and pea protein microgels (PPM) can stabilize O/W emulsions containing 20 wt% sunflower oil [31]. Emulsions with PPM alone were unstable at pH 3, leading to droplet coalescence within 30 min. Adding CNC particles improved stability through interfacial electrostatic interactions on the outer side of the PPM layer between anionic CNC and positively charged PPM particles. A gel-like structure was generated at higher CNC concentrations, attributed to CNC network formation that increased bulk viscosity.

## HYPIES BASED ON THE SYNERGY BETWEEN ONE ‘HARD’ OR ‘SOFT’ PARTICLE AND A SURFACTANT

The combination of hydrophilic particles and molecular surfactants—including cationic, anionic, zwitterionic and non-ionic surfactants—provides a straightforward approach to generate HYPIEs. Surfactants can modify particle wettability at the oil–water interface, promote particle adsorption, or induce local repulsions that fix particles at the interface. Both synthetic and biobased surfactants (e.g. sugar-derived) are employed, with the latter traditionally used in food formulations.

### HYPIEs based on the synergy between one particle and a cationic surfactant

#### Synergy between one anionic ‘hard’ particle and a cationic surfactant

Negatively charged silica or alumina particles interact synergistically with cationic surfactants to stabilize emulsions. The electrostatic attraction enhances particle adsorption at the oil–water interface. Such HYPIEs may partially agglomerate, increasing particle size and improving interfacial stability. This approach also allows design of stimuli-responsive emulsions controlled by pH, temperature, redox potential or magnetic fields.

**Figure 4.**
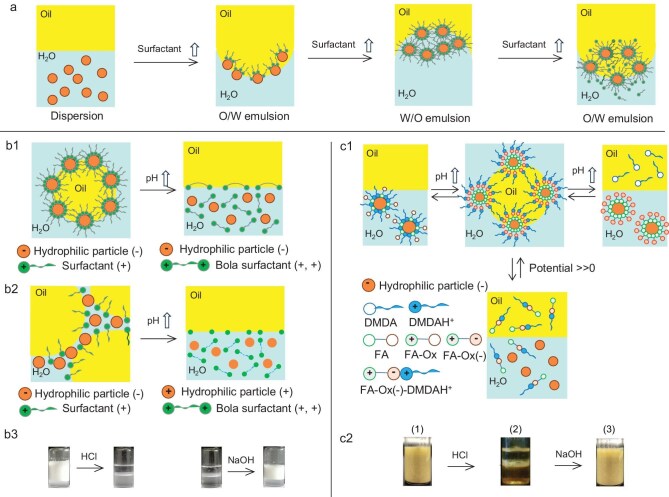
(a) Interaction between anionic silica particles and cationic surfactants, promoting flocculation with the surfactant concentration and in turn emulsion conversion [32]. Emulsification performance of (b1) anionic silica particles and (b2) cationic alumina particles combined with pH-responsive trimethylammonium bromide [N^+^–(*n*)–N, *n* = 14, 16] surfactants [33]. (b3) Optical images of O/W emulsions stabilized by pH-responsive trimethylammonium bromide undergoing demulsification and re-stabilization at pH 2.5 and pH 10.7, respectively (reproduced from Pei *et al.* [33] with permission from Wiley). (c1) Emulsification properties of anionic silica particles combined with redox, pH-responsive FA–DMDA–Ox surfactant synthesized *in situ* via neutralization of FA with DMDA [36]. (c2) Optical images of emulsion droplets stabilized by silica particles and DMDA after adding HCl and NaOH, alternately (reproduced from Li *et al*. [36] with permission from the American Chemical Society).

Silica particles combined with quaternary amines such as cetyltrimethylammonium bromide (CTAB) below the critical micelle concentration (CMC) (0.9 mM) exhibit enhanced hydrophobicity, reduced interfacial tension and suppressed droplet coalescence [32]. Near-neutral particle charge promotes flocculation, generating highly stable emulsions (Fig. 4a). Above the CMC, surfactant layers adsorb on particle surfaces, preventing coalescence. Pei and coworkers [33] reported alkyl trimethylammonium bromide [N^+^–(*n*)–N; *n* = 14, 16] surfactants that stabilized emulsions differently depending on the pH: neutral/alkaline media produced O/W emulsions with oppositely charged silica and oil-in-dispersion (O/dispersion) emulsions with similarly charged particles (e.g. cationic aluminas) (Fig. 4b1 and b2), while acidic conditions transformed the surfactant into a hydrophilic bola-type surfactant, N^+^–(*n*)–NH^+^, inducing demulsification. This form returned to the aqueous phase without contaminating the oil, and aqueous phase recycling through pH adjustment.

Silicas and aluminas can be combined with a cationic surfactant bearing electroactive heads to create redox-responsive interfaces. For example, 11-ferrocenylundecyltrimethylammonium bromide (FcCOC10N) at 0.01 mM can synergize with silica particles to yield long-term stable emulsions [34]. Similarly, alumina particles combined with ferrocene surfactants capable of switching between single- and double-head forms (FcN + C12/Fc + N + C12) stabilized O/dispersion emulsions at ultralow surfactant and particle concentrations (0.01 mM and 0.001 wt%, respectively) [35]. In contrast, the single-head surfactant FcN + C12 was unable to stabilize emulsions with alumina particles due to steric hindrance from the ferrocene group.

Tertiary amine surfactants combined with anionic silica particles can generate pH- and redox-responsive HYPIEs. Li *et al.* [36] combined silica particles with the redox- and pH-responsive surfactant FA–DMDA–Ox (0.1 wt%), synthesized via neutralization of ferrocenecarboxylic acid (FA) with *N,N*-dimethyldodecylamine (DMDA) (Fig. 4c). Demulsification–emulsification was controlled by alternating additions of Na_2_SO_3_ and H_2_O_2_, promoting reversible adsorption–desorption of FA–DMDA–Ox on silica particles. The emulsion was switched ‘off’ by adding HCl and ‘on’ by adding NaOH, driven by controlled dispersion of silica particles and FA–DMDA–Ox.

Liu *et al.* [37] reported O/W emulsions based on diesel oils stabilized with silica and *N*-(2-((2-aminoethyl)amino)ethyl)octadecenamide (C_18_PDA) surfactant with secondary amine and primary groups in the same molecule, in the presence of Na_2_CO_3_. At low surfactant concentrations, increasing salt levels reduces silica–C_18_PDA attraction due to enhanced hydration repulsion, enhancing hydrophilicity and promoting O/W emulsions. By adjusting the surfactant concentration, silica-stabilized emulsions underwent phase inversion between O/W (10–20 mM C_18_PDA, 94 mM salt) and W/O (above 20 mM, 94 mM salt), or vice versa at lower salt concentrations (<20 mM). At higher concentrations, silica particles partially dispersed with reduced flocculation, leading to emulsion destabilization.

Hydrophilic silica combined with *N′*-dodecyl-*N,N*-dimethylacetamidine (DDMA) can produce CO_2_/N_2_-switchable HYPIEs due to *in situ* partial hydrophobization via electrostatic interactions. CO_2_ converted DDMA into the amidinium bicarbonate form, stabilizing emulsions, while bubbling N_2_ or air reversed it to the inactive amidine form destabilizing emulsions [38].

Combining cationic and anionic surfactants with silica particles was reported to enable reversible emulsion stabilization [39]. CTAB initially stabilized O/W emulsions by *in situ* hydrophobization, while subsequent addition of sodium dodecyl sulfate (SDS) formed CTAB–SDS ion pairs, destabilizing emulsions. Re-addition of CTAB restored stability. Re-addition of CTAB with rehomogenization restored the emulsion. Xu *et al.* [40] demonstrated CO_2_/N_2_-switchable emulsions using alumina particles with SDS and DDMA surfactants. The hydrophilic alumina particles were *in situ* hydrophobized by adsorbing SDS on their surface. Upon bubbling CO_2_, DDMA switched to its cationic amidinium form that generated ion pairs with SDS molecules, causing emulsion destabilization.

Weakly surface-active cationic electrolytes (e.g. *R*_4_N^+^X^−^, *R* = C_1_–C_4_) can promote the interfacial adsorption of anionic silica particles, stabilizing 1-octane/W emulsions at low salt concentrations (≥0.05 mM) without particle flocculation [41,42]. Stability is governed by electrostatic repulsions between droplets and between droplets and particles.

Zeolites can be combined with cationic surfactants to build HYPIEs stabilizing emulsions. High-internal phase emulsions were stabilized by A-type zeolite with tetradecyltrimethylammonium bromide (TTAB) adsorbing on particle surfaces via electrostatic interactions [43]. Low TTAB/zeolite ratios (≤0.2 wt%) produced gel-like O/W high-internal phase emulsions, while intermediate ratios induced phase inversion via hydrophobization, and high ratios (>1.25 wt%) induced a second phase inversion generating creamy O/W emulsions through TTAB bilayer formation on particles. Droplet size and network strength were governed by the zeolite particle concentration, where excess particles agglomerated, creating a secondary network of flocs in the continuous phase interconnected with particles adsorbed at the oil–water interface.

Montmorillonite (MMT) combined with C_12_–C_14_ alkylamine ethoxylate chloride (Berol R648) can generate highly stable emulsions, with NaCl promoting flocculation and generating a mechanical barrier around droplets with particles oriented parallel to the droplet surface [44]. MMT combined with bis(2-hydroxyethyl)oleylamine (E-O/12) stabilized W/O emulsions with seawater at 0.1 wt% MMT [45]. Emulsions inverted to O/W either by decreasing the surfactant-to-MMT weight ratio (transitional inversion) or increasing the water volume fraction (catastrophic inversion). Tetramethylammonium chloride (TMAC)/laponite systems stabilized alkenyl succinic anhydride (ASA)/O emulsions through particle charge neutralization by TMAC adsorption on the particles, generating a dense network that increased continuous phase viscosity [46]. This improved emulsion stability against creaming and significantly reduced droplet size. Whitby *et al.* [47] stabilized Pickering emulsions with 30-nm disc-shaped laponite particles. Flocculation-induced micrometer-sized aggregates formed in the presence of octadecylamine (ODA), stabilizing O/W emulsions, while antagonistic interactions with octadecanoic acid caused droplet coalescence. The synergy at the oil–water interface reduced the average droplet size from 220 μm without surfactant to 60 μm with 1 mM ODA.

HYPIEs based on positively charged alumina particles and cationic DDMA surfactant can stabilize decane/W emulsions at ultralow DDMA concentrations (0.004 CMC) [48]. Without particles, emulsions stabilized solely by low surfactant concentrations were unstable due to low interfacial surfactant adsorption such that coalescence was prevalent. When positively charged alumina particles were added, they dispersed evenly in the aqueous phase. Double-layer repulsion between particles and between particles and charged droplets generated thick lamellae that increased the minimum distance between droplets and reduced van der Waals attraction between droplets favoring emulsion stabilization. A critical zeta potential of *ca*. +18 mV was necessary for emulsion formation, highlighting the role of electrostatic double-layer repulsions in stabilization.

CNC particles can be modified *in situ* with small amounts of didecyldimethylammonium bromide (DMAB) or CTAB [49]. Below the CMC, surfactants adsorbed with tails outward, increasing hydrophobicity of CNC particles, while above the CMC, surfactant aggregates formed on CNC particles reducing hydrophobization. DMAB, with two alkyl tails, imparted higher hydrophobicity than CTAB at the same concentration. Changes in CNC wettability due to surfactant adsorption were directly linked to emulsion properties: surfactant addition enhanced emulsion stability, reduced droplet size and controlled the internal volume. Increasing DMAB concentration caused a double phase transition (O/W to W/O, followed by W/O to O/W). In contrast, CNC particles modified with CTAB did not induce phase inversion.

### HYPIEs based on the synergy between a particle and an anionic surfactant

#### Synergy between one ‘hard’ particle and an anionic surfactant

**Figure 5.**
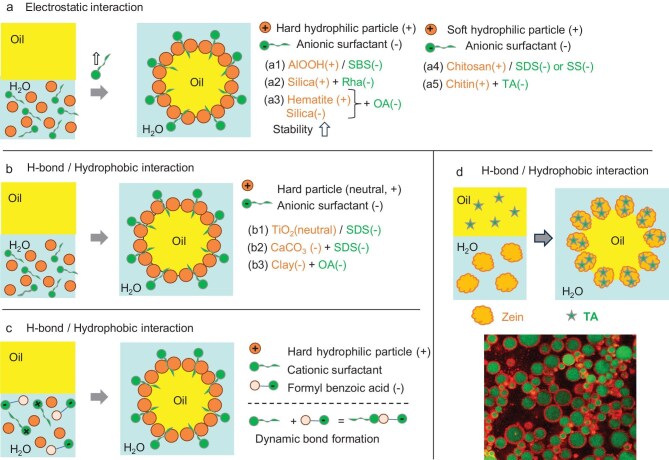
HYPIEs based on cationic ‘hard’ or ‘soft’ particles combined with an anionic surfactant or fatty carboxylic acid driven by electrostatic interactions: (a1) AlOOH (hard, +) and SBS (−), (a2) silica (hard, +) and Rha (−), (a3) hematite (hard, +), silica (hard, −) and OA (−), (a4) CS (soft, +) and SDS (−) or SS (−), (a5) chitin (soft, +) and TA (−). Images adapted from Jia *et al.* [50], Pi *et al*. [51] and Tiwari *et al.* [52] (hard particles) and from Ren and Zhang [60] and Wang *et al.* [61] (soft particles). HYPIEs based on a neutral or anionic ‘hard’ particle combined with an anionic surfactant or fatty carboxylic acids driven by hydrogen-bond or hydrophobic interactions: (b1) TiO_2_ (hard, neutral) and SDS (−); (b2) CaCO_3_ (hard, −) and SDS (−); (b3) clay (hard, −) and OA (–) [53,56–58]. (c) HYPIEs based on anionic silica particles and an *in situ*-generated anionic surfactant through dynamic bond formation [59]. (d) HYPIEs based on zein particles and TA prepared by an antisolvent method [64], and confocal laser scanning microscopy image of an emulsion stabilized by zein particles and TA (protein is stained in red and oil, in green) (reproduced from Zou *et al.* [64] with permission from the American Chemical Society).

HYPIEs can be engineered combining positively charged particles with anionic surfactants through electrostatic interactions. For example, sodium benzenesulfonate (SBS) displays monolayer adsorption on flake-like hydrophilic AlOOH particles, increasing both the particle hydrophobicity and viscosity of dispersions, forming a 3D network in the continuous phase and preventing droplet coalescence (Fig. 5a1) [50]. Emulsions were resistant to shear, temperature and electrolytes, making them suitable for EOR. Positively charged silica particles combined with the anionic biobased surfactant rhamnolipid (Rha) stabilized emulsions in seawater through partial flocculation and steric barriers, producing smaller droplets than single-component systems (Fig. 5a2) [51]. *In situ* adsorption of Rha on silica partially flocculated the particles at the oil–water interface and imparted a steric barrier preventing droplet coalescence. Adding oleic acid (HOA) to O/W emulsions stabilized by anionic silica and cationic hematite induced the formation of O/W/O double emulsions for the decane/W system, with the droplet size being controlled by HOA concentration and the specific surface area of the particles (Fig. 5a3) [52]. Emulsion formation was attributed to *in situ* hydrophobization of silica–hematite dual assemblies at the decane/W interface. The critical concentration of HOA required to form a double emulsion varied with the ratio of the surface area of the silica particle to the total surface area of particles.

HYPIEs can be generated by pre-adsorbing SDS on neutral TiO_2_ particles (isoelectric point) driven by hydrophobic interactions, generating silicone/W emulsions at SDS concentrations below 1/50 CMC (Fig. 5b1) [53]. Flocculated TiO_2_ formed a 3D network, imparting yield stress and viscoelasticity. HYPIEs between colloidal particles and anionic surfactants can also be formed through preferential hydrogen-bonding at the oil–water interface. Whitby *et al.* [54] studied 1-dodecane/W emulsions stabilized by partially hydrophobized silica particles in the presence of SDS and NaCl. Emulsions were resistant to coalescence upon dilution in salt solutions but exhibited creaming over time as salt concentration increased. At SDS concentrations above the CMC, both creaming and flocculation rates increased. Santini *et al.* [55] prepared W/1-hexane emulsions stabilized by hydrophilic silica particles combined with palmitic acid. *In situ* adsorption of palmitic acid on silica can form dense, persistent layers preventing coalescence. Very stable emulsions can be obtained with a single palmitic acid layer on the particle surface.

Examples have been reported on HYPIEs combining anionic particles and surfactants. Addition of trace amounts of SDS to an aqueous suspension of CaCO_3_ particles generated stable O/W emulsions (Fig. 5b2) [56]. Increasing the SDS concentration caused a double phase inversion: from O/W(1) to (W/O), and then from W/O to O/W(2). SDS adsorption on the particles rendered them hydrophobic, triggering the first inversion. At higher SDS concentrations, bilayer formation on the particles restored their hydrophilicity, causing the second inversion. HYPIEs were also engineered combining hydroxyapatite or kaolinite particles with sodium oleate or HOA (Fig. 5b3) [57,58]. The particles exhibited an antagonistic effect with the surfactant concentration forming W/O emulsions that rapidly broke down. This instability was attributed to the adsorption of nearly all surfactant molecules on the particle surfaces, causing hydrophobic particles to transfer from the aqueous to the oil phase without interfacial adsorption. This occurred due to the peeling of the second oleate molecule layer due to competitive adsorption between the interface and saturated particle surface.

Emulsions can be stabilized by HYPIEs based on the assembly of negatively charged silica particles and an *in situ*-generated anionic surfactant though dynamic bond formation (e.g. imines from 4-formylbenzoic acid and hexylamine) (Fig. 5c) [59]. Emulsions formed at surfactant concentration below the CMC and 0.5 wt% silica particles. Repulsive interactions dominated, and pH-controlled bond formation allowed reversible emulsion stabilization/destabilization.

#### Synergy between one ‘soft’ particle and an anionic surfactant

Anionic surfactants can synergize with ‘soft’ particles with positively charged surface groups to formulate emulsions driven by electrostatic interactions. For example, CS did stabilize emulsions under acid conditions due to a high water wettability caused by protonated ammonium groups (Fig. 5a4) [60]. However, a trace amount of SDS (approximately 1/100 to 1/10 of CMC) interacted electrostatically with ammonium groups, increasing CS hydrophobicity and altering the interfacial conformation and activity. As a result, high-molecular-weight CS precipitated as microsized flocs, while low-molecular-weight CS transformed into a non-covalent polymer surfactant. The resulting emulsions rapidly destabilized by adding CTAB, causing SDS to desorb from CS and react with CTAB. Emulsions were reversibly switched ‘on/off’ using SDS and CTAB. Wang *et al.* [61] combined cationic ‘soft’ chitin particles with tannic acid (TA), producing physically and oxidatively stable O/W emulsions (Fig. 5a5).

Proteins such as zein can interact with anionic surfactants via electrostatic interactions and hydrogen-bonding, enhancing interfacial adsorption, particle unfolding and close packing at the interface. Gao *et al.* [62] combined colloidal zein particles with sodium stearate (SS), resulting in enhanced interfacial adsorption and accumulation of zein particles as SS concentration increased. Partial unfolding of zein particles modified by SS above its critical complexation concentration triggered by aggregation and close packing at the interface, provided a steric barrier against oil droplet coalescence for emulsification. Likewise, Cui *et al.* [63] combined zein particles with *Quillaja* saponin (QS), forming a dense 6.6-nm thick interfacial coating that altered zein’s interfacial properties and improved emulsion stability.

HYPIEs have been designed for the formulation of edible emulsions formed from zein particles and TA driven by hydrogen-bonding (Fig. 5d) [64]. Using an antisolvent approach and hydrogen-bond interactions between zein and TA, near-neutral HYPIEs with enhanced interfacial reactivity were formed. This interaction triggered interfacial crosslinking between HYPIEs, forming a continuous network between oil droplets and resulting in stable emulsion gels. Zou *et al.* [65] combined zein protein particles of different sizes (68 and 108 nm) with TA by adjusting the precursor suspension pH. The resulting HYPIEs stabilized O/W high-internal phase Pickering emulsion gels containing 72–85 wt% oil at three different charge densities (+38, +20, +1 mV) using 0.7–1.4 wt% particle concentration. Smaller particle sizes reduced the oil content in the gels due to the formation of a particle network in the continuous phase, resulting in higher storage modulus. Gel strength was further increased by lowering the charge density and thus particle interactions. Notably, gel strength was independent of oil content, indicating oil droplets contributed little to gel rigidity.

### HYPIEs based on the synergy between a particle and a zwitterionic surfactant

Zwitterionic surfactants can form HYPIEs combined with particles through electrostatic and hydrogen-bond interactions, enhancing emulsion stability and responsiveness. Reported examples involve typically ‘hard’ particles and surfactants with varied molecular structures. Worthen *et al.* [66] stabilized O/W emulsions combining silica particles and caprylamidopropyl betaine, containing carboxylate and quaternary amine groups. The surfactant primarily adsorbed at the oil–water interface, lowering interfacial tension, while silica particles acted as steric barriers against coalescence. The resulting HYPIEs produced smaller droplets and reduced creaming rates compared to particle- or surfactant-only systems. Fu *et al.* [67] developed reversible, CO_2_/N_2_-responsive HYPIEs combining silica with a supramolecular amphiphile (SDOA) from polyetheramine (e.g. JEFFAMINE D-230) and HOA at a 1:1 molar ratio. The O/W emulsions remained stable for over 90 days at a low surfactant concentration (0.5 mM) through *in situ* hydrophobization of silica particles driven by hydrogen-bonding and electrostatic adsorption. Introducing CO_2_ lowered the pH, decomposing the pseudo-amphiphile and destabilizing the emulsion within 10 s. Subsequent N_2_ purging at 60°C for 5 min restored stability, regenerating the amphiphilic complex, allowing repeated, reversible switching of emulsion stability.

### HYPIEs based on the synergy between a particle and a non-ionic surfactant

#### Synergy between one ‘hard’ particle and a non-ionic surfactant

**Figure 6.**
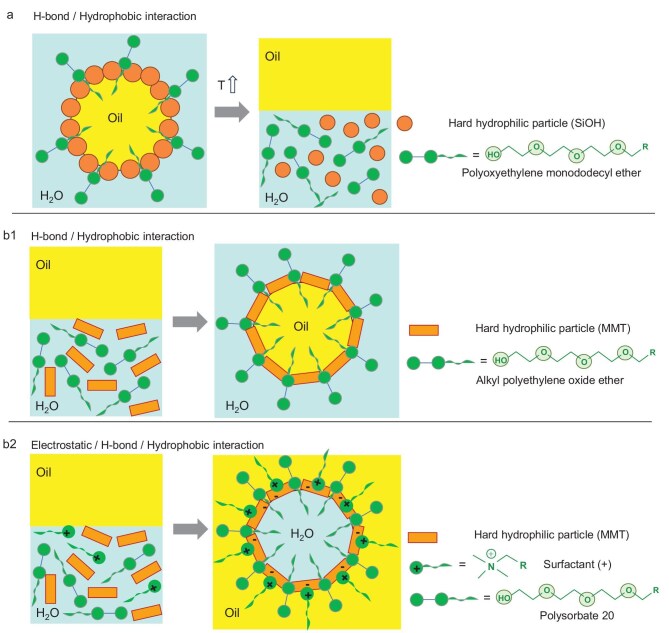
HYPIEs based on anionic ‘hard’ particles combined with a non-ionic surfactant driven by hydrogen-bond interactions: (a) thermoresponsive emulsions stabilized by HYPIEs based on silicas combined with polyoxyethylene monododecyl ether [68]. (b1) O/W emulsions stabilized by MMT combined with alkyl polyethylene oxide ethers [70]. (b2) W/O emulsions stabilized by MMT combined with stearyl trimethylammonium chloride and polysorbate 20 [72].

Non-ionic surfactants such as alkyl polyoxyethylene glycol monomers (e.g. C_12_EO_7.8_) can adsorb on silica particles through hydrogen-bonding, inducing *in situ* hydrophobization. Combining hydrophilic silica with polyoxyethylene monododecyl ether (C_12_E_n_) at low concentration yielded thermoresponsive O/W emulsions that were stable at room temperature but reversibly destabilized upon heating (45–65°C) by weakening of hydrogen bonds (Fig. 6a) [68]. Demulsification rate and transition temperature depended on surfactant headgroup length, reflecting temperature-dependent weakening of hydrogen bonds.

Lv *et al.* [69] demonstrated a synergy between hollow TS-1 zeolite particles and sorbitan monooleate (Span 80) at the oil–water interface, forming stable W/O emulsions of cyclohexanone and water. Increasing Span 80 concentration (0.5–8 mM) for 2 wt% TS-1 induced phase inversion by altering its adsorption within TS-1 mesopores, affecting interfacial layer thickness and particle–surfactant interactions.

Non-ionic surfactants exhibit strong synergy with clays. Lagaly *et al.* [70] stabilized emulsions using MMT combined with glycerol monostearate, alkyl polyethylene oxide ethers and alkyl polyglycosides (Fig. 6b1). Adsorbed MMT platelets enhanced interfacial mechanical strength and reduced electrostatic repulsion at the oil–water interface, with organophilic platelets reducing weak electrostatic repulsions between particles and strengthening the mechanical stability of the interface. Nallamilli and Basavaraj [71] studied 1-decane/W emulsions stabilized by kaolinite particles combined with Span 80. No stable emulsions formed below 3 mM, while stable W/O emulsions appeared above 5 mM. At 0.3 wt% kaolinite concentration, no emulsions formed at Span 80 concentrations below 3 mM. Between 3 and 7 mM, O/W emulsions were unstable, whereas stabilization was promoted at 10–30 mM. Phase inversion from O/W to W/O occurred above 30 mM, attributed to competitive adsorption of excess Span 80 on droplets, while bilayer-covered particles remained dispersed.

Opposing previous studies leading to O/W emulsions, Yang *et al.* [72] produced W/O nanoemulsions by combining stearyl trimethylammonium chloride (STAC)-modified MMT with non-ionic surfactants (Fig. 6b2). The most stable emulsions formed when STAC–MMT was added to the oil phase (vitamin E and mineral oil), and the surfactant to the aqueous phase (aqueous phase/emulsifier/oil phase ratio of 1:1:8, w/w), followed by slow addition of water to oil.

‘Hard’ CNF particles combined with polyoxyethylene (20) sorbitan monooleate (polysorbate 80) form vegetable O/W emulsions where CNFs create a rigid interfacial network [73]. Adding surfactant decreased droplet size but slightly lowered network stiffness.

Recent studies highlight HYPIEs combining natural organic particles and non-ionic surfactants for food applications. Zein–polysorbate 20 systems displayed *synergistic-competitive* stabilization [74]. At low surfactant levels (≤1 wt%), zein–polysorbate aggregates promoted interfacial adsorption and emulsion stability, while at higher levels (≥1.5 wt%), competition displaced zein from the interface. Dissipative particle dynamics (DPD) simulations confirmed this shift (4.5 → 10 Å), linking stability to controlled interfacial aggregation. The promoted interfacial adsorption of zein particles at low polysorbate 20 concentrations was linked to inhibition of interfacial Brownian motion.

## HYPIES BASED ON THE SYNERGY BETWEEN ONE PARTICLE AND AN UNFOLDED POLYMER

HYPIEs can be engineered by combining particles with unfolded (extended) polymers, which—unlike compact globular polymers—offer greater surface area and accessible functional groups, promoting hydrophobic and hydrogen-bonding interactions with particles at the oil–water interface. When adsorbed at the oil–water interface, these polymers form thick steric barriers that enhance stability and reduce interfacial tension.

### HYPIEs based on the synergy between one particle and an unfolded synthetic polymer

#### Synergy between one ‘hard’ particle and an unfolded synthetic polymer

**Figure 7.**
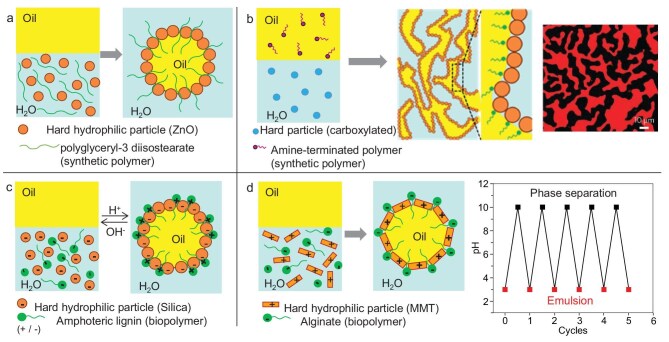
HYPIEs based on particles and unfolded polymers. (a) Formation of O/W emulsions (O = mefentrifluconazole) by adsorption of polyglyceryl-3 diisostearate on ZnO particles driven by hydrogen-bonding [75]. (b) Formation of bijels using polystyrene particles combined with amine-terminated PDMS [78], and confocal laser scanning microscopy image of bijel (reproduced from Huang *et al.* [78] with permission from Springer). (c) pH-responsive mechanism of emulsions stabilized by HYPIEs based on hydrophilic silica particles and AML. At pH 3–4, positively charged AML molecules adsorb electrostatically onto negatively charged silica. Increasing pH leads to phase separation due to both negatively charged AML and silica [80]. (d) Emulsions stabilized by HYPIEs based on hydrophilic sodium MMT with ALG molecular chains [82] (reproduced from Wang *et al.* [82] with permission from Elsevier).

Zheng *et al.* [75] prepared a stable 3% mefentrifluconazole emulsion containing 70 vol% water phase using flower-like ZnO (0.5 wt%) and polymer A-7 (mainly polyglyceryl-3 diisostearate) (0.1 wt%), which co-adsorbed at the oil–water interface to form dense layers preventing coalescence (Fig. 7a). The emulsion rapidly inhibited droplet rebound and deposited effectively on rice leaves with different inclination angles within 1.5 ms, spreading to a maximum wetting area within 360 s. A dual-responsive (pH and magnetic) emulsion was designed using Fe_3_O_4_ particles combined with poly(2-(dimethylamino)ethyl methacrylate) (PDMAEMA), allowing reversible emulsification and demulsification [76]. The hydrophobicity of PDMAEMA increased significantly at higher pH due to deprotonation of surface amine groups.

Wang *et al.* [77] stabilized paraffin/W emulsions using laponite particles modified *in situ* with poly(oxypropylene)diamine. Anchored polymer end groups enhanced surface hydrophobicity, while exposed poly(oxypropylene) chains improved dispersion and interfacial stabilization.

Huang *et al.* [78] formed bicontinuous jammed emulsions (bijels) using hydrophilic carboxylated polystyrene particles in water and hydrophobic amine-terminated polydimethylsiloxane (PDMS) in oil (Fig. 7b). Particles jammed at the oil–water interface created submicron channels (<10 μm). This approach enables bijel formation across diverse particle–polymer–solvent systems.

#### *In situ* generation of a particle by interaction between a large anion and an unfolded synthetic polymer

Interactions between molecular compounds and particles can induce particle self-assembly, producing macroscopic supramolecular entities or supracolloids through colloidal tectonics—a bottom-up approach based on spontaneous formation of supracolloidal structures from molecular building blocks called ‘tectons’. Pacaud *et al.* [79] demonstrated self-assembled colloidal systems using this strategy by introducing PW_12_O_40_^3−^ anions into inclusion complexes formed by β-cyclodextrin (β-CD) and oil molecules, promoting emulsification. Depending on concentration, these complexes formed spherical particles or microcrystals, which were used to design catalytic systems leveraging the activity of PW_12_O_40_^3−^ anions.

### HYPIEs based on the synergy between one particle and an unfolded biopolymer

Biopolymers derived from renewable sources (proteins, polysaccharides) are attractive for biodegradable and biocompatible emulsions, though their hydrophilicity limits interfacial activity. Combining them with particles offers a simple route to wettability control without chemical modification.

#### Synergy between one ‘hard’ particle and an unfolded biopolymer

Lu *et al.* [80] developed pH-responsive HYPIEs via *in situ* hydrophobization of silica particles using amphoteric lignin (AML). Electrostatic adsorption between AML positively charged backbone and negatively charged silica enhanced hydrophobicity and emulsion stability at pH 3–4 (Fig. 7c). At pH > 4, strong electrostatic repulsions lead to unstable emulsions. Pi *et al.* [81] stabilized O/W emulsions with seawater using a mixture of xanthan gum (XG) and silica particles. This HYPIE increased the continuous-phase viscoelasticity and formed thick droplet coatings, yielding smaller, more stable droplets even at low particle loadings.

HYPIEs can form directly at the oil–water interface through combinations of clay particles and unfolded biopolymers. Wang *et al.* [82] combined hydrophilic sodium MMT with alginate (ALG) molecular chains to create stable emulsions (Fig. 7d). Hydrogen-bonding and electrostatic interactions between ALG and MMT created a gel-like interfacial network, enhancing dispersion and resistance to coalescence.

Zhou *et al.* [83] formed HYPIEs by pre-adsorbing medium-chain triglyceride (MCT) onto zein particles through hydrogen-bonding and hydrophobic interactions. Increasing the zein/MCT ratio exposed more hydrophobic groups, accelerating interfacial diffusion and rearrangement, and forming a viscoelastic interfacial network resistant to deformation. This network strengthened interparticle interactions, improving emulsion responsiveness and resistance to deformation under external pressure, thus enhancing emulsion stability. Wang and Heuzey [84] designed HYPIEs based on CS/gelatin B complex particles stabilizing O/W emulsions at pH 5–6 through electrostatic interactions. Emulsions had smaller droplet sizes and greater long-term stability compared to those stabilized by soluble complexes due to stronger protective interfacial barriers. Increasing oil fraction promoted droplet network formation, yielding thermoreversible, solid-like emulsions gel with tunable viscoelasticity.

## APPLICATION OF HYPIEs

HYPIEs thus provide a tunable platform for smart catalytic emulsions, extendable to photocatalysis, biocatalysis, EOR and biomedical applications.

### Catalysis

#### Chemocatalysis

Pickering interfacial catalysis (PIC) enhances biphasic reactions by improving mutual contact and mass transfer between substrates of opposite polarity [85]. Emulsions provide large interfacial areas and local miscibility relative to bulk reactions, enabling tandem and cascade reactions under biphasic conditions. Multifunctional catalysts can promote tandem or cascade reactions by combining multiple reactions into a single process. Tandem reactions can be carried out over two surface-active particles with different catalytic sites within emulsions. Through the colloidal tectonics concept, different catalytic particles can self-assemble at the oil–water interface for cooperative catalysis.

**Figure 8.**
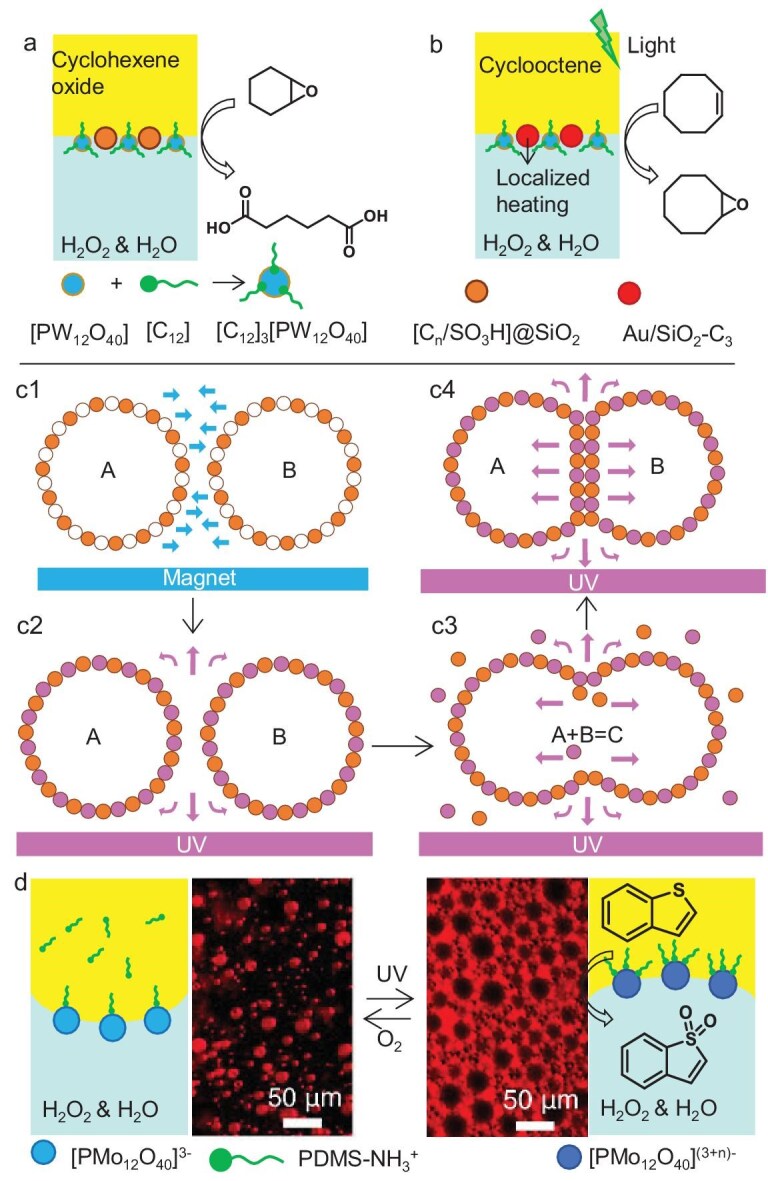
(a) Catalytic tandem reaction for adipic acid synthesis using [C_18_/SO_3_H]@SiO_2_ and [C_12_]_3_[PW_12_O_40_] particles as catalyst and emulsifier driven by partial penetration of alkyl chains of [C_18_/SO_3_H]@SiO_2_ into [C_12_]_3_[PW_12_O_40_] [86]. (b) Schematic representation of emulsion stabilized by self-assembled amphiphilic silica particles loaded with Au nanoparticles, acting as on-site heater/plasmon activators, and [C_12_]_3_[PW_12_O_40_] particles, acting as catalyst, under UV light irradiation [87]. (c) Coalescence mechanism for the stabilization of Pickering emulsions by titania and magnetite HYPIEs [88]. (d) Schematic illustration of POM functionalized with PDMS–NH_2_ polymer to generate light/O_2_-triggered reversible emulsion inversion [89], and optical images of emulsions formed (reproduced from Wan *et al.* [89] with permission from the American Chemical Society).

Yang *et al.* [86] designed HYPIEs combining dodecyltrimethylammonium phosphotungstate particles ([C_12_]_3_[PW_12_O_40_]) and silica particles functionalized with alkyl and sulfonic acid groups ([C_*n*_/SO_3_H]@SiO_2_) to catalyze the one-pot oxidative cleavage of cyclohexene oxide to adipic acid using aqueous H_2_O_2_ in emulsion (Fig. 8a). Interlocking of C_18_ chains from [C_18_/SO_3_H]@SiO_2_ within the porous phosphotungstate structure of [C_12_]_3_[PW_12_O_40_] particles reduced droplet size and improved emulsion stability. In a related system, [C_12_]_3_[PW_12_O_40_] particles were combined with plasmonic Au@SiO_2_-C_n_/SH particles modified with alkyl/mercaptopropyl groups, acting as both interfacial catalysts and on-site photo-assisted heaters/activators (Fig. 8b) [87]. UV irradiation increased oxidation activity 5-fold compared to thermal catalysis at 50°C, achieving up to 74% energy savings and excellent recyclability compared to conventional reactors.

Xie *et al.* [88] reported light- and magnetically responsive W/O emulsions stabilized by partially hydrophobized titania and hydrophilic magnetite particles. Van der Waals interactions enabled co-adsorption at the interface, forming a liquid oil film between particle layers (Fig. 8c). An external magnetic field induced droplet contact, while UV light altered titania wettability to trigger particle detachment, film drainage and on-demand reactant release for acid–base or precipitation reactions.

Wan and coworkers [89] prepared emulsions stabilized by polyoxometalate (POM)–PDMS–NH_2_ interfacial co-assemblies via dynamic electrostatic interactions (Fig. 8d). The number of polymer chains anchored to POMs tuned their wettability, further modulated by *in situ* redox reactions that altered POM charge. This enabled light- and oxygen-triggered emulsion inversion for cyclic oxidative desulfurization of benzothiophene, allowing efficient product separation and catalyst reuse.

#### Photocatalysis

Two recent examples of HYPIEs have been reported for applications in photocatalysis. Shi *et al.* [90] engineered Rha-modified TiO_2_ HYPIEs with enhanced oil spill dispersion and photocatalytic degradation capacity. Rhamnolipid improved TiO_2_ wettability and interfacial packing, forming rigid interfacial layers around oil droplets in artificial seawater. Li *et al.* [91] stabilized O/W emulsions using unmodified TiO_2_ particles. Adsorption of methyl orange on the TiO_2_ surface imparted hydrophobicity, stabilizing emulsions. The emulsions enhanced photocatalytic dye degradation compared to biphasic systems without emulsions.

#### Biocatalysis

Bio-HYPIEs enable enzyme localization at the oil–water interface, enhancing mass transfer and activating lipases by ‘lid’ opening. Enzymes can interact with particles and proteins during emulsification. Yu and coworkers [92] prepared CO_2_/N_2_-responsive emulsions stabilized by silica particles hydrophobized *in situ* with DMDA, containing *Candida rugosa* lipase (CRL) in the aqueous phase. Hydrogen-bonding and hydrophobic interactions enhanced hydrolysis and esterification, with demulsification and enzyme recovery triggered by CO_2_/N_2_ cycling. Seiler *et al.* [93] prepared O/W emulsions stabilized by positively or negatively charged silica particles combined with *Candida antarctica* lipase A (CALA). The enzyme activity optimum shifted depending on particle charge: acidic pH (5) for negatively charged particles; and neutral pH for positively charged ones. CALA adsorption altered particle hydrophobicity and emulsion stability. Xi and coworkers [94] developed CO_2_/N_2_-responsive O/W emulsions stabilized by NaCas, in which sodium caseinate is combined with CALB to form NaCas-CALB HYPIEs. CO_2_/N_2_ cycling enabled product separation and >90% yield retention over 30 reuse cycles.

### EOR

Pickering emulsions are applied in oil recovery under high-temperature and high-pressure conditions, offering improved flooding efficiency and reduced carbon footprint. HYPIEs based on combinations of particles and surfactants can improve emulsion rheology by lowering interfacial tension and modifying porous media wettability. Recent advances focus on HYPIE-based nanofluids, utilizing negatively charged silica and polymer particles with cationic or non-ionic surfactants.

#### Nanofluids based on particle–surfactant HYPIEs

Pei *et al.* [95] utilized negatively charged silica particles hydrophobized with CTAB, enhancing emulsion stability and flooding efficiency. The silica–CTAB synergy reduced particle concentration in the aqueous phase, lowering emulsion viscosity. The silica–CTAB synergy reduced particle dosage and emulsion viscosity, performing effectively in heavy oil reservoirs with permeability in the range 500–2000 mD and crude oil viscosity of <1000 mPa·s. Lee and Babadagli [96] employed silica particles with DTAB and alcohol propoxy sulfate (ALFOTERRA S23-7S-90), generating *in situ* emulsions at the pore scale and achieving 27% higher oil recovery compared to brine flooding. Zhao *et al.* [97] used glucose-based non-ionic surfactant (GBDD)-modified halloysite nanotubes, where increasing GBDD concentration caused emulsion inversion (O/W → W/O → O/W). The nanofluid enhanced oil detachment and reduced flow resistance through wedge adsorption effects, improving recovery efficiency.

#### Nanofluids based on particle–polymer HYPIEs

Zhang *et al.* [98] adsorbed poly(ethylene oxide)PEO–poly(propylene oxide)PPO–PEO triblock copolymer onto silica particles via hydrogen-bonding, forming pH- and thermo-responsive paraffin O/W emulsions. Low pH and room temperature induced silica flocculation and hydrophobization, while high pH or temperature restored hydrophilicity and dispersion.

#### Nanofluids based on particle–surfactant–polymer HYPIEs

**Figure 9.**
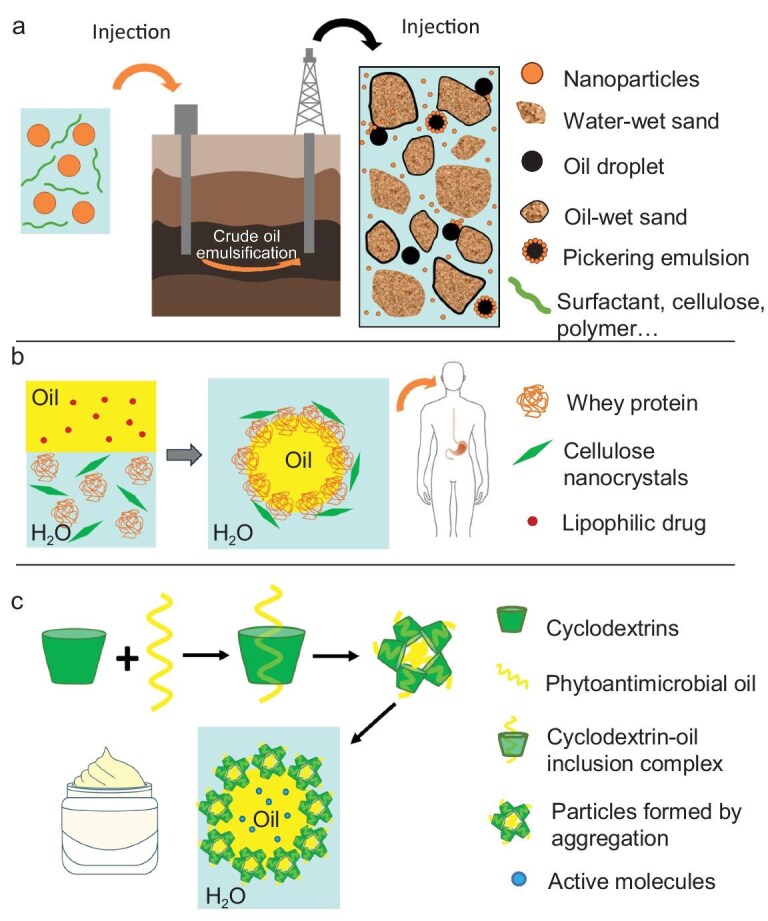
(a) Schematic illustration of oil recovery mechanism with nanofluid flooding facilitating oil droplet detachment [99]. (b) Schematic representation of emulsion stabilized by whey proteins and cellulose nanocrystals loaded with lipophilic drug for biomedicine applications [101]. (c) Schematic illustration of emulsions stabilized by HYPIEs formed by CD–oil inclusion complex to load active molecules for cosmetics applications [108].

Kumar *et al.* [99] demonstrated synergistic effects between silica and carboxymethyl cellulose (CMC) particles, and anionic surfactant sodium dodecylbenzene sulfonate (SDBS), lowering the interfacial tension by ∼10% and reducing droplet size (3.66 vs. 7.25 μm) (Fig. 9a). Injection of 0.5 pore volume yielded 24% improvement in oil recovery using 0.25 wt% silica, 1.5 wt% CMC and 825 ppm SDBS.

### Biomedicine

HYPIEs have attracted attention in biomedical fields for stabilizing biocompatible emulsions and enabling controlled delivery of active compounds. Systems include particle–surfactant, two-particle and two-particle + surfactant combinations.

#### HYPIEs based on silica–surfactant combinations

Sihler *et al.* [100] prepared inverse emulsions stabilized by silica particles and surfactants, forming robust droplet shells and 3D internal networks through *in situ* hydrophobization via van der Waals and hydrogen-bonding interactions, achieving excellent stability and narrow size distribution.

#### HYPIEs based on two-particle combinations

Whey protein isolate (WPI) and CNCs stabilized emulsions at pH 3, where optimized CNC levels created dense interfacial layers that limited pepsin access and reduced proteolysis kinetics (Fig. 9b) [101]. Chen *et al.* [102] developed W/O/W double emulsions using CS/soy β-conglycinin complexes and algal oil for bioactive delivery. Optimal stability occurred at a CS/7S ratio of 1:2 and 40 wt% oil, with particle contact angles near 90°.

#### HYPIEs based on two-particle + surfactant combinations

Liu *et al.* [103] prepared zein–tannic acid (TA)–ALG HYPIEs, suitable for β-carotene encapsulation and 3D food-grade printing, due to favorable viscoelastic and rheological properties.

### Cosmetics and personal care products

HYPIEs are emerging as sustainable stabilizers for cosmetic and dermatological formulations, offering enhanced delivery, stability and skin compatibility. Sadeghpour *et al.* [104] used HYPIEs with HOA-coated silica particles to stabilize hydrophobic oils with nanoscale droplets (∼100 nm). Sharkawy *et al.* [105] synthesized CS–gum Arabic particles with tunable wettability for cosmetic emulsions, improving resveratrol and cannabidiol protection and photostability. Xu *et al.* [106] employed octenyl succinic anhydride starch–CS HYPIEs for topical resveratrol delivery, achieving 6-fold enhancement in skin uptake.

CD complexes are promising in the design of HYPIEs for application in cosmetics owing to their ability to form host–guest inclusion complexes. Mathapa and Paunov [107] prepared CD-stabilized emulsions using various oils as continuous phase, including high-viscosity silicone oil. The size of CDs was a key parameter for emulsion stabilization. CDs formed cyclodextrinosomes by removing the oil from the emulsion. These structures were stable in water and exhibited significant potential as carriers for active molecules in cosmetic applications. Leclercq and Nardello-Rataj [108] used CD-based HYPIEs with phytoantimicrobial oils (carvacrol, terpinen-4-ol) and econazole nitrate (Fig. 9c), achieving potent antimicrobial and antifungal performance. A patented formulation [109] describes CD-based emulsions stabilized by a complex of CDs and natural-based emulsifiers, including alkyl polyglucosides, mixtures of alkyl polyglucosides and fatty alcohols, or non-ethoxylated polyol fatty esters, specifically for temperature-sensitive cosmetic formulations.

## CONCLUSIONS AND PERSPECTIVES

**Figure 10.**
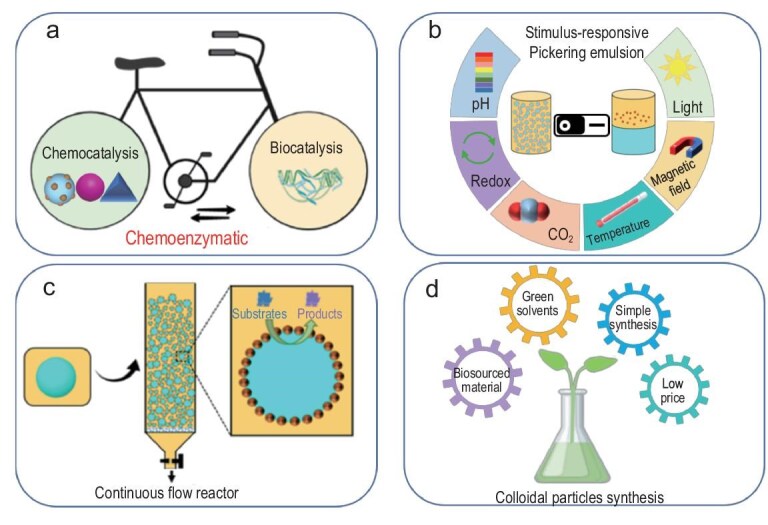
Perspectives of HYPIEs for designing chemoenzymatic reactions in Pickering emulsion (a), stimuli-responsive Pickering emulsions (b), Pickering emulsions in continuous flow (c) and colloidal particle design (d).

In this review, we have summarized the recent progress in the design of hybrid particle–interface emulsions (HYPIEs), where synergistic interactions between particles and co-emulsifiers at the oil–water interface yield functionalities unattainable with particles alone. HYPIEs can be created via *in situ* modification using surfactants or polymers through electrostatic and hydrogen-bond interactions, or by assembling supracolloidal structures via colloidal tectonics. Future opportunities lie in expanding HYPIEs toward chemoenzymatic catalysis, stimuli-responsive and flow-type emulsions, as well as computationally guided colloidal design (Fig. 10). In the cosmetics sector, HYPIEs offer controlled release of active ingredients, improved product performance, and sustainability by replacing petrochemical emulsifiers with natural or hybrid biocolloids, aligning with the growing demand for cleaner-label, sustainable cosmetic formulations.

### Chemoenzymatic and tandem catalysis in Pickering emulsion

Integrating chemocatalysis and biocatalysis in one-pot HYPIE systems enables cascade reactions that minimize solvent use, energy consumption and waste generation. Since chemocatalysis typically occurs in organic media and biocatalysis in aqueous media, HYPIEs combining catalytic particles and enzymes at the interface can bridge this incompatibility, providing efficient, green routes to multistep synthesis (Fig. 10a).

### Stimuli-responsive Pickering emulsion

The development of switchable emulsions—capable of emulsification/demulsification or phase inversion upon external stimuli—offers versatile control for applications in pharmaceuticals, petroleum processing and catalyst recovery (Fig. 10b). Light, temperature or magnetic fields can modulate surface wettability without additives. Future designs may focus on multi-responsive hybrid particles integrating thermo-, photo- and magneto-sensitivity to precisely regulate emulsion behavior in complex environments.

### Pickering emulsion in continuous flow

Most reported Pickering emulsion systems operate in batch mode, which limits their scalability, reproducibility and industrial relevance. Continuous-flow processing represents a critical step toward the practical implementation of HYPIE-based catalytic systems, offering advantages such as enhanced mass and heat transfer, precise residence-time control, and straightforward catalyst recovery and product separation (Fig. 10c). However, translating Pickering emulsions into flow reactors introduces new challenges, particularly with respect to maintaining emulsion integrity under shear, pressure fluctuations and prolonged operation.

A key obstacle lies in the mechanical robustness of interfacial particle films, which must withstand continuous flow without droplet coalescence, phase inversion or particle detachment. In this context, hybrid particle–interface architectures provide unique opportunities to reinforce interfacial layers through synergistic interactions between particles and co-emulsifiers, such as polymers or surfactants, thereby enhancing resistance to shear and chemical stress. Rationally designed HYPIEs could enable long-term stable emulsification, sustained catalytic activity and minimized fouling in flow environments.

Future research should focus on integrating HYPIEs with microfluidic platforms to achieve controlled droplet generation, scalable throughput and modular reactor design. In parallel, coupling continuous emulsification with in-line separation and recycling strategies will be essential for realizing closed-loop catalytic processes.

### Colloidal particle design

Various types of particles have been employed as stabilizers for formulating Pickering emulsions. Among them, biobased particles and Janus particles have attracted considerable attention due to their environmentally friendly nature and multifunctionality. However, biobased particles often require time-consuming pretreatments, which can generate organic waste, while Janus particles typically involve complex synthesis procedures to meet specific application requirements. In recent years, particle preparation processes have become increasingly intricate, as chemists tend to remain within familiar approaches, sometimes overlooking the value of simplicity and the principle of ‘less is more.’ We believe that the development of Pickering emulsions should move toward simplified particle pretreatments, a reduced environmental footprint and tailored interfacial properties (Fig. 10d). In this context, the next generation of Pickering emulsions, stabilized by two or more types of particles, offers the potential to design systems with enhanced stability and improved functionalities compared to conventional emulsions. Further exploration of the synergistic benefits provided by dual or multiple particle combinations holds great promise for advancing green and sustainable chemistry.

### *In silico* design of HYPIEs

*In silico* design of HYPIEs using computational methods can provide valuable information for predicting emulsion stability and rationalizing particle location and film architecture at the oil–water interface. To this end, a multiscale modeling approach—combining quantum mechanics (density functional theory; DFT), classical or coarse-grained molecular dynamics (MD), dissipative particle dynamics (DPD) and machine learning (ML)—can offer a comprehensive framework for predicting and optimizing particle interactions and HYPIE formation at oil–water interfaces since Pickering emulsions are composed of microscale droplets, nanoscale HYPIEs based on particles, folded polymers and proteins, and molecules (surfactants, unfolded proteins). The synergy between these computational techniques enables both detailed mechanistic understanding and predictive capabilities, ultimately guiding the rational design of emulsifiers, stabilizers and functional interfacial materials.

DPD is a mesoscopic simulation technique suitable for modeling complex fluids and soft matter. It is especially valuable for predicting the collective behavior of polymers, surfactants and nanoparticles at interfaces over longer timescales. DPD can simulate self-assembly, interfacial tension reduction and the stabilization mechanisms of emulsions, enabling the study of how macromolecules or mixed surfactant systems adsorb onto particles at the oil–water interface and modify the interfacial behavior. However, most studies to date have focused on single particles with minor attempts to study HYPIEs.

Classical MD is widely used to study the behavior of molecules and particles at interfaces. It can capture atomic-level interactions between particles (e.g. nanoparticles, colloids) and amphiphilic molecules such as surfactants or proteins. MD allows the modeling of adsorption dynamics, interfacial structuring and the thermodynamics of binding. Force fields like CHARMM, GROMOS, OPLS-AA or MARTINI (for coarse-grained MD) are often employed depending on the system’s complexity and resolution required. The synergistic effects of non-ionic surfactants and particles have also been explored in atomistic MD simulations. Recent advances in MD incorporate complex oils (e.g. aromatic hydrocarbons, ionic liquids) and functional additives (e.g. surfactant–polymer combinations or switchable surfactants), bringing simulation conditions closer to experimental reality. Nonetheless, limitations remain, particularly with respect to time and length scales, restricting the direct modeling of long-term coalescence or large-scale emulsion behavior.

Developing a fully automated, computation-driven method to determine optimal synergistic interactions based on existing simulation or experimental data to design HYPIEs, while modeling the effects of various parameters (particle nature, size, surfactant/polymer type, interaction type…) that would impact the emulsion properties (stability, droplet size…) remains highly challenging. ML models can be trained to predict adsorption energies, preferred configurations or interfacial tension changes as a function of molecular descriptors or system composition. These models could assist the design of tailored HYPIEs and identify the most effective combination of particles, surfactants, polymers and proteins to predict the adsorption behavior of particles at the oil–water interface and their influence on the stability, emulsion properties and properties for target applications.
